# Adipocytokine Profiling and Cardiovascular Risk Stratification in Rheumatoid Arthritis: Literature Review

**DOI:** 10.3390/jcm15218445

**Published:** 2026-10-29

**Authors:** Abdalla Elsayed, Abdulrahman Ismaiel, Mihai Bojinca

**Affiliations:** 1 Faculty of Medicine, Carol Davila University of Medicine and Pharmacy, 050474 Bucharest, Romania; mihai.bojinca@umfcd.ro; 2 Internal Medicine and Rheumatology Department, Cantacuzino Clinical Hospital, 020475 Bucharest, Romania; 3 2nd Department of Internal Medicine, “Iuliu Hatieganu” University of Medicine and Pharmacy, 400006 Cluj-Napoca, Romania

**Keywords:** rheumatoid arthritis, cardiovascular risk, adipokines, visfatin, meta-inflammation

## Abstract

Rheumatoid arthritis (RA) is associated with an increased burden of cardiovascular disease (CVD), reflecting interactions among persistent systemic inflammation, traditional cardiometabolic risk factors, and treatment exposure. Conventional risk scores may underestimate cardiovascular risk in some patients with RA. This narrative review examines the potential role of adipokines in linking synovial inflammation and metabolic dysfunction to vascular disease. It discusses the context-dependent cardiovascular associations of adiponectin, the pro-inflammatory signaling of leptin, resistin, visfatin, and chemerin, and the proposed protective roles of omentin, vaspin, and apelin. Emerging observations connect adipokine profiles with measures of subclinical atherosclerosis and selected clinical outcomes; however, methodological heterogeneity and residual confounding constrain causal and prognostic interpretations. We also consider venous thromboembolism, cardiometabolic multimorbidity, and treatment-related risk, including the clinical safety context of JAK inhibitors. At present, adipokine profiling remains investigational and should complement research, not replace established cardiovascular risk assessment and control of disease activity and conventional risk factors.

## 1. Introduction

Rheumatoid arthritis (RA) is a chronic, systemic autoimmune disease characterized not only by synovial inflammation and articular destruction but also by a profound alteration in systemic metabolism and vascular biology. It is now well-established that patients with RA experience a 1.5- to 2-fold increase in the risk of cardiovascular disease (CVD) compared to the general population, a mortality gap that has persisted despite improvements in antirheumatic therapies [1]. This excess risk is largely attributable to accelerated atherosclerosis, which manifests clinically as myocardial infarction (MI), stroke, and heart failure (HF). Crucially, traditional cardiovascular risk scores, such as the Framingham Risk Score or the Systematic Coronary Risk Evaluation (SCORE), consistently underestimate the actual event rate in RA patients, often by as much as 30% to 50% [2]. This diagnostic blind spot necessitates the identification of novel biomarkers that can capture the specific pathogenic intersections between autoimmunity, inflammation, and vascular injury.

Importantly, RA should not be regarded as an exclusively articular disorder. Persistent systemic inflammation contributes to extra-articular manifestations, including pulmonary, cardiovascular, and other organ involvement, with consequences for prognosis and therapeutic decision-making. As discussed by Conforti et al., these manifestations reflect the systemic nature of RA and may coexist with an elevated burden of conventional cardiovascular risk factors [3]. Cumulative inflammatory exposure, reflected by sustained disease activity and inflammatory markers, may interact with hypertension, diabetes mellitus, dyslipidemia, obesity, and smoking to promote vascular injury. Accordingly, cardiovascular assessment in RA should address both conventional determinants of risk and RA-specific inflammatory characteristics.

In the past decade, the scientific focus has shifted from the “lipid paradox,” where low total cholesterol and low-density lipoprotein (LDL) levels are paradoxically associated with higher cardiovascular risk due to inflammation-driven catabolism, to the role of adipose tissue as an active endocrine organ. Far from being a passive energy depot, adipose tissue is a dynamic source of bioactive peptides known as adipokines. These molecules act as potent regulators of appetite, glucose homeostasis, and, critically, immune responses and vascular tone [4].

In the context of RA, the dysregulation of adipokines creates a milieu of “adipo-inflammation.” Synovial fibroblasts, infiltrating macrophages, and endothelial cells within the inflamed joint and systemic vasculature express receptors for these adipokines, creating autocrine and paracrine loops that perpetuate inflammation and endothelial dysfunction. While early research focused heavily on resistin and visfatin due to their clear pro-inflammatory properties, recent investigations have elucidated the nuanced and often paradoxical roles of a broader spectrum of adipokines, including adiponectin, leptin, chemerin, omentin, vaspin, nesfatin-1, progranulin, and apelin [5]. This review provides a narrative synthesis of the literature, detailing the pathophysiological mechanisms, predictive utility, and therapeutic modulation of these molecules in the context of RA cardiovascular risk.

### 1.1. The Cardiovascular Phenotype in RA

The cardiovascular phenotype of RA differs from that of the general population. It is characterized by plaque instability, microvascular dysfunction, and a higher incidence of heart failure with preserved ejection fraction (HFpEF), driven by myocardial stiffness and fibrosis [2]. The inflammatory cytokine storm, dominated by tumor necrosis factor-alpha (TNF-α), interleukin-6 (IL-6), and interleukin-1 (IL-1), promotes endothelial activation and oxidative stress. Adipokines serve as the bridge between this inflammatory signaling and metabolic substrates. For instance, while obesity is a traditional risk factor for CVD in the general population, in RA, a “bimodal” risk pattern is often observed where both cachexia (wasting) and obesity confer risk. Adipokines like adiponectin and leptin fluctuate wildly between these states, serving as biomarkers for specific metabolic-inflammatory phenotypes [6].

Cardiovascular involvement in RA extends beyond atherosclerotic events. Venous thromboembolism (VTE), encompassing deep-vein thrombosis and pulmonary embolism, is an additional clinically relevant outcome. In a real-world RA cohort, Conforti et al. documented VTE events and identified older age, higher body mass index, and longer RA duration as associated factors [7]. VTE and atherosclerotic cardiovascular disease have overlapping but nonidentical determinants; conventional atherosclerotic risk algorithms should therefore not be interpreted as validated tools for estimating VTE risk.

The coexistence of cardiometabolic conditions is also important. Ruscitti et al. assessed 757 consecutive patients with RA and defined cardiometabolic multimorbidity as at least two of hypertension, dyslipidemia, and type 2 diabetes; 13.5% met this definition [8]. Compared with those without cardiometabolic multimorbidity, affected patients more frequently exhibited extra-articular manifestations, were less frequently in remission, and had a more frequent history of biological disease-modifying antirheumatic drug (bDMARD) failure. These cross-sectional associations are consistent with a clinically more complex RA phenotype, although they do not establish that cardiometabolic multimorbidity causes more severe RA.

These clinical observations align with EULAR recommendations that emphasize optimal control of inflammatory disease activity together with periodic cardiovascular risk assessment and management of modifiable risk factors in inflammatory arthritis [9]. The potential contribution of adipokines to this broader framework is discussed below.

### 1.2. Adipose Tissue as an Immune Organ

**Figure 1:**
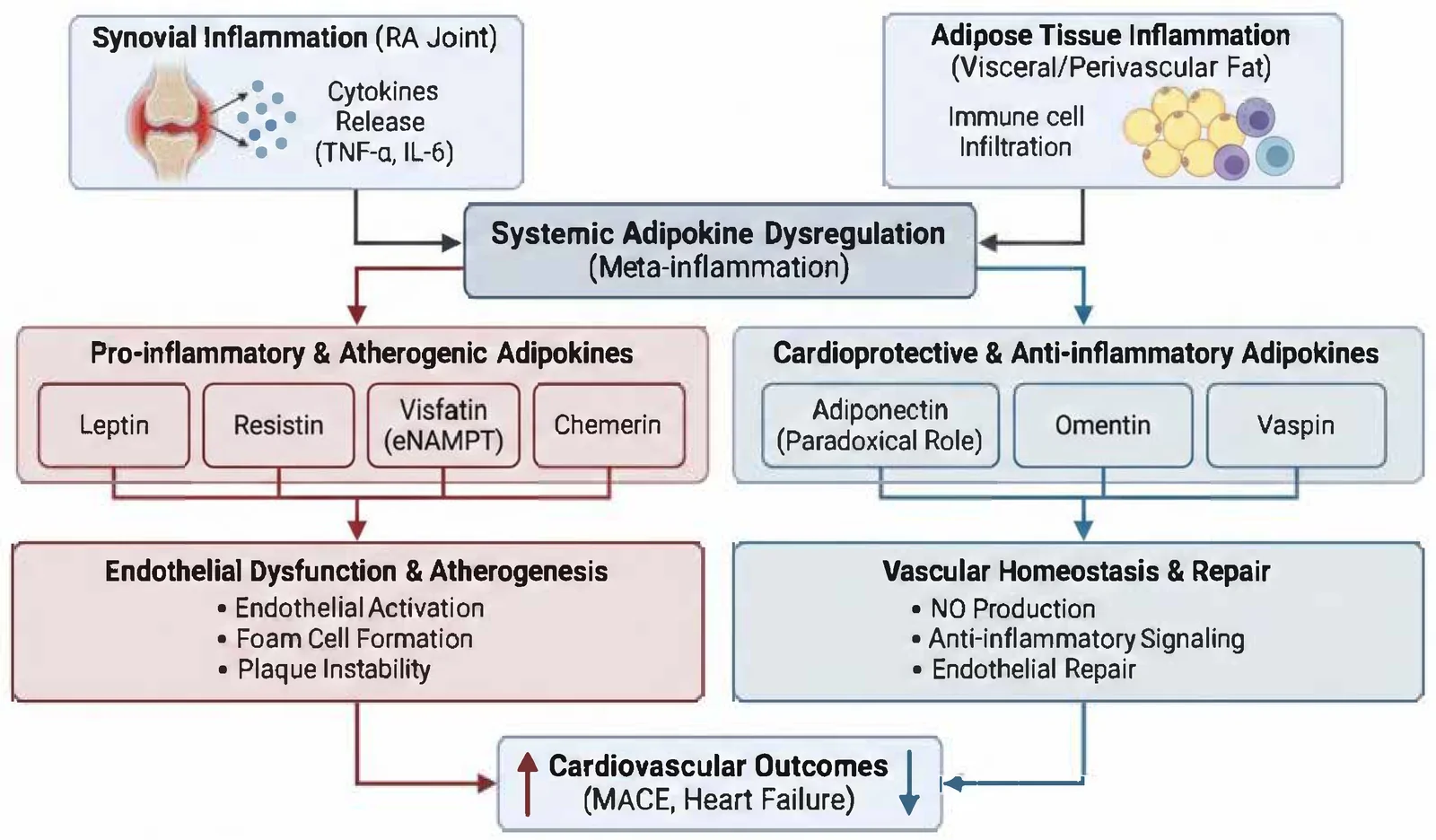
Proposed mechanism of adipokine-mediated cardiovascular risk in rheumatoid arthritis.

The concept of the “adipose–immune–vascular axis” is central to understanding modern RA pathophysiology. As outlined in Figure 1, several mechanisms play a role in the adipokine-mediated cardiovascular risk in rheumatoid arthritis. In RA, adipose tissue undergoes qualitative changes, often infiltrated by M1-polarized macrophages that secrete pro-inflammatory adipokines. Furthermore, the synovium in RA shares histological features with inflamed adipose tissue, and synoviocytes themselves are capable of secreting adipokines such as adiponectin and chemerin, which then act locally to destroy cartilage and systemically to impair vascular relaxation [10]. Understanding the specific contribution of each adipokine allows for a more granular risk stratification that moves beyond simple lipid panels. Table 1 summarizes the role of adipokines in RA cardiovascular risk.

## 2. The Adiponectin Paradox: A Critical Re-Evaluation of Cardioprotection

Adiponectin is the most abundant adipokine in human plasma and is canonically regarded as a cardioprotective, insulin-sensitizing, and anti-inflammatory molecule in the general population. It inhibits the transformation of macrophages into foam cells, suppresses the expression of adhesion molecules (ICAM-1, VCAM-1) on endothelial cells, and promotes nitric oxide (NO) production via the AMP-activated protein kinase (AMPK) pathway [11]. However, in severe chronic inflammatory diseases like RA and heart failure, this relationship is inverted, leading to the phenomenon known as the “Adiponectin Paradox.”

### 2.1. Epidemiological Evidence of the Paradox

Recent large-scale longitudinal studies have provided robust evidence that in RA, high circulating levels of adiponectin are associated with increased, rather than decreased, all-cause and cardiovascular mortality. A seminal study from the Veterans Affairs Rheumatoid Arthritis (VARA) registry (2023) analyzed the association of adipokines with CVD outcomes in a large cohort of RA patients. The investigators found that high adiponectin levels were independently associated with a significantly higher risk of heart failure (HF) hospitalization (Hazard Ratio 1.39; 95% CI 1.07–1.79) and CVD-related death (HR 1.49; 95% CI 1.16–1.92) [11].

This association persists even after adjusting for traditional risk factors, suggesting that in the RA context, hyperadiponectinemia reflects a distinct pathological process. The association was noted to be strongest in non-obese patients, lending weight to the hypothesis that elevated adiponectin in RA is a marker of “rheumatoid cachexia,” a state of metabolic wasting driven by high-grade inflammation, where loss of fat mass and muscle mass is accompanied by a paradoxical upregulation of adiponectin [11]. In this scenario, adiponectin acts as a distress signal of catabolic stress rather than a protective buffer.

### 2.2. Isoform-Specific Risks: High vs. Low Molecular Weight

Total adiponectin measurement may obscure the distinct biological activities of its multimeric isoforms. Adiponectin circulates in three primary forms: low-molecular-weight (LMW) trimers, medium-molecular-weight (MMW) hexamers, and high-molecular-weight (HMW) octadecamers. HMW Adiponectin is generally considered the biologically active, beneficial isoform responsible for insulin sensitization and vascular protection. On the other hand, LMW Adiponectin isoforms in the context of RA appear to have pro-inflammatory properties.

Research indicates that the ratio of these isoforms is critical. While HMW adiponectin may remain neutral or protective, elevated levels of LMW adiponectin have been specifically linked to all-cause mortality (HR 1.13) in RA cohorts [21]. The disruption of the enzymatic machinery in the endoplasmic reticulum required to assemble HMW multimers (due to inflammatory stress) may shift the secretory profile toward the LMW form, explaining why total levels rise while vascular protection fails [22].

### 2.3. The NT-proBNP Confounder

A pivotal mechanistic insight is the interaction between adiponectin and natriuretic peptides. N-terminal pro-B-type natriuretic peptide (NT-proBNP) is a gold-standard marker for myocardial stretch and heart failure. Basic science and clinical correlations have demonstrated that natriuretic peptides stimulate the secretion of adiponectin from adipocytes [23].

In RA patients with subclinical heart failure or myocardial stiffness, conditions highly prevalent in this population, NT-proBNP levels rise, driving a secondary increase in adiponectin. When statistical models do not adjust for NT-proBNP, adiponectin appears to “cause” mortality. However, when models are adjusted for NT-proBNP, the association between adiponectin and mortality is often attenuated, suggesting that adiponectin is, at least in part, a bystander or a downstream marker of cardiac strain [23]. This potential confounding is important for clinicians; a high adiponectin level in an RA patient should trigger an evaluation for underlying heart failure and volume overload rather than reassurance of metabolic health.

### 2.4. Mechanisms of Vascular Damage in RA

Beyond its role as a marker, adiponectin may exert direct deleterious effects on the RA vasculature. In the presence of synovial fibroblasts and inflammatory cytokines, adiponectin has been shown to induce the expression of Endocan, a proteoglycan marker of endothelial activation, and VEGF (Vascular Endothelial Growth Factor) via MEK/ERK signaling pathways [1]. This promotion of pathological angiogenesis contributes to both the invasive nature of the pannus in the joint and the destabilization of atherosclerotic plaques in the vessel wall. Thus, the “Dr. Jekyll and Mr. Hyde” nature of adiponectin is context-dependent: protective in the metabolic syndrome, but potentially destructive in the autoimmune joint and vasculature.

## 3. Leptin: The Pro-Inflammatory Driver and Thrombotic Risk

Leptin, the hormone of energy expenditure and satiety, is structurally homologous to IL-6 and acts as a potent pro-inflammatory cytokine. It is a critical regulator of the immune system, promoting the differentiation of naïve T cells into pro-inflammatory Th1 and Th17 lineages while suppressing regulatory T cells (Tregs) [24]. In RA, serum leptin levels are consistently elevated and correlate with disease activity, making it a prime candidate for cardiovascular risk prediction.

### 3.1. Association with Cardiovascular Mortality and VTE

Unlike adiponectin, the cardiovascular risk associated with leptin follows a more direct, albeit BMI-dependent, trajectory.

High leptin levels are independently associated with CVD-related mortality (HR 1.44; 95% CI 1.05–1.97) in RA patients. Crucially, this association is driven by non-obese patients [11]. In obese patients, “leptin resistance” (downregulation of Ob-R receptors) may blunt the systemic signaling effects. However, in non-obese RA patients, elevated leptin relative to fat mass indicates a disproportionate inflammatory drive, leading to worse outcomes.

RA patients have a well-documented increase in VTE risk. Leptin has emerged as a significant independent predictor of this risk (HR 1.26; 95% CI 1.05–1.52) [11]. Mechanistically, functional leptin receptors are expressed on platelets. Leptin stimulation sensitizes platelets to agonists like ADP and collagen, lowering the threshold for aggregation. Furthermore, leptin upregulates Plasminogen Activator Inhibitor-1 (PAI-1) in endothelial cells, impairing fibrinolysis and promoting a pro-thrombotic state [2].

Venous thromboembolism (VTE) requires clinical consideration distinct from atherosclerotic cardiovascular disease. Real-world observations in RA, including those reported by Conforti et al., support VTE as a relevant clinical complication in the setting of chronic systemic inflammation [7]. Associations of leptin with platelet activation and impaired fibrinolysis provide a plausible mechanistic hypothesis; however, evidence linking individual adipokine concentrations to VTE is substantially less developed than evidence for the overall association between RA and VTE. No individual adipokine has been sufficiently validated to guide routine VTE screening, anticoagulant prophylaxis, or treatment decisions.

### 3.2. Mechanisms of Atherogenesis

Leptin contributes to the atherosclerotic process in RA through multiple convergent pathways. Leptin induces the production of reactive oxygen species (ROS) in endothelial cells, depleting nitric oxide bioavailability and impairing vasodilation. Moreover, chronic hyperleptinemia increases sympathetic nervous system tone. Since hypertension is highly prevalent and often poorly controlled in RA, leptin serves as a metabolic driver of neurogenic hypertension [4]. Furthermore, leptin stimulates the migration and proliferation of vascular smooth muscle cells (VSMCs), a key step in arterial stiffening and plaque formation.

### 3.3. Mendelian Randomization and Causality

While observational data strongly link leptin to CV outcomes, recent Mendelian Randomization (MR) studies (2023) have sought to determine causality regarding the onset of RA. These studies found no evidence that genetically determined leptin levels directly cause RA after adjusting for BMI [25]. This distinction is vital: leptin may not be the initiator of the autoimmune disease, but once the disease is established, it acts as a powerful amplifier of the cardiovascular comorbidities associated with it.

## 4. The Chemerin–Omentin Axis: Novel Biomarkers of Endothelial Dysfunction

As research expands beyond the classical adipokines, the balance between chemerin and omentin has emerged as a sophisticated indicator of endothelial health. The “chemerin/omentin ratio” is increasingly investigated as a superior predictor of subclinical atherosclerosis than either marker alone.

### 4.1. Chemerin: The Chemotactic Agitator

Chemerin is a chemoattractant protein that binds to the ChemR23 (CMKLR1) receptor, which is expressed on dendritic cells, macrophages, and endothelial cells. In RA, chemerin is highly expressed in the synovium and correlates with systemic inflammation.

Chemerin acts directly on the endothelium to upregulate adhesion molecules (ICAM-1, E-selectin) via p38 MAPK and NF-κB pathways, facilitating the recruitment of inflammatory cells to the vessel wall [26]. Moreover, serum chemerin levels are significantly elevated in RA patients compared to controls and correlate positively with C-reactive protein (CRP), erythrocyte sedimentation rate (ESR), and DAS28 scores [1].

High chemerin levels have been identified as an independent risk factor for ischemic stroke and have been proposed as a marker of early endothelial dysfunction in essential hypertension [27]. In RA cohorts, elevated chemerin is associated with unstable atherosclerotic plaque phenotypes and increased arterial stiffness [16].

Furthermore, the rs17173608 G allele of the chemerin gene is associated with increased susceptibility to RA and higher circulating chemerin levels, suggesting a genetic predisposition to this “adipo-inflammatory” phenotype [20].

### 4.2. Omentin: The Protective Factor

Omentin (Intelectin-1) is unique among adipokines as it is preferentially produced by the stromal vascular fraction of visceral adipose tissue, and its expression is downregulated in states of obesity and inflammation. It exerts cardioprotective effects by activating AMPK in endothelial cells, which leads to the phosphorylation of endothelial nitric oxide synthase (eNOS) at Ser1177, enhancing nitric oxide production and vasodilation [28]. It also inhibits TNF-α-induced vascular inflammation.

Omentin levels are consistently lower in RA patients compared to healthy controls, inversely correlating with inflammatory markers [16]. This depletion removes a critical “brake” on vascular inflammation.

A strong negative correlation has been documented between serum omentin levels and subclinical atherosclerosis, as represented by increased carotid intima-media thickness (cIMT) in patients with RA and axial spondyloarthritis [16]. Low omentin is an independent predictor of arterial stiffness and the presence of carotid plaques.

Treatment with TNF inhibitors (e.g., adalimumab, etanercept) has been shown to restore omentin levels. This restoration correlates with improvements in flow-mediated dilation (FMD), suggesting that omentin recovery is a marker of successful vascular risk reduction [29].

## 5. Resistin and Visfatin: The Pro-Inflammatory Core

While much attention has been paid to adiponectin and leptin, resistin and visfatin (NAMPT) have emerged as specific markers of “meta-inflammation”—the intersection of metabolic dysregulation and high-grade immunity. New evidence provides granular detail on their role in plaque vulnerability and heart failure.

### 5.1. Resistin: The Synergistic Risk Marker

Resistin, primarily secreted by macrophages in humans (distinct from its adipocyte origin in rodents), acts as a direct link between the immune system and vascular dysfunction. It binds to Toll-like receptor 4 (TLR4) and Adenylate cyclase-associated protein 1 (CAP1) on endothelial cells. This binding triggers the NF-κB signaling cascade, upregulating adhesion molecules (VCAM-1, ICAM-1) and promoting the infiltration of monocytes into the arterial wall [30]. Furthermore, resistin directly impairs cardiomyocyte function by inhibiting the cardioprotective AMP-activated protein kinase (AMPK) pathway and promoting cardiac fibrosis via TGF-β signaling [30]. A pivotal study stratified RA patients by the presence of metabolic syndrome (MetS). The authors found that serum resistin levels were highest in RA patients with MetS, intermediate in RA patients without MetS, and lowest in healthy controls. Crucially, resistin levels correlated strongly with Carotid Intima-Media Thickness (CIMT) (r = 0.446, *p* < 0.001). This identifies resistin as a biomarker of the additive burden of autoimmunity and metabolic adiposity [12].

While some markers predict ischemic events, resistin is a potent predictor of heart failure. In longitudinal cohorts adjusted for traditional risk factors, higher resistin levels were significantly associated with incident Heart Failure (HR 1.44; 95% CI 1.18–1.75) and specifically Heart Failure with Reduced Ejection Fraction (HFrEF) (HR 1.47) [31]. This aligns with its mechanistic role in inducing myocardial fibrosis and hypertrophy [30].

### 5.2. Visfatin (NAMPT): The “Erosion Paradox” and Plaque Instability

Visfatin, also known as Nicotinamide Phosphoribosyltransferase (NAMPT), functions as a dual-edged molecule: intracellularly (iNAMPT) as an essential enzyme for NAD+ biosynthesis, and extracellularly (eNAMPT) as a pro-inflammatory cytokine. It is heavily expressed in macrophage-rich regions of unstable atherosclerotic plaques. It promotes the secretion of Matrix Metalloproteinases (MMP-2, MMP-9), which degrade the fibrous cap of the plaque, increasing the risk of rupture and acute coronary syndromes (ACS). Clinical studies confirm that high visfatin levels are independently associated with Major Adverse Cardiovascular Events (MACE) in patients with acute myocardial infarction [14].

A recent study unveiled a complex relationship between visfatin and joint damage. While serum visfatin levels are generally elevated in RA compared to controls, lower serum visfatin levels were unexpectedly associated with a higher prevalence of joint erosions [12]. The authors postulate a “local sequestration” hypothesis: during active erosive disease, circulating visfatin is consumed or sequestered within the inflamed synovium to fuel the high metabolic demand (NAD+ salvage) of proliferating pannus cells, leading to deceptively lower serum levels despite high local activity [12]. Despite this paradox in joint erosions, elevated circulating visfatin remains positively correlated with endothelial dysfunction and CIMT, confirming its systemic vasculotoxic effects [12].

## 6. Emerging Adipokines: Vaspin, Nesfatin-1, Progranulin, and Apelin

The “second wave” of adipokine research in RA (2020–2026) has focused on characterizing less common molecules that may offer specific insights into myocardial and vascular pathology.

### 6.1. Vaspin (Visceral Adipose Tissue-Derived Serpin)

Vaspin is a serine protease inhibitor that generally functions as an insulin-sensitizing adipokine. Contrary to expectations for a “protective” molecule, vaspin levels are often elevated in RA synovial fluid and serum compared to osteoarthritis and healthy controls [20]. This is interpreted as a compensatory mechanism: the body upregulates vaspin in an attempt to counteract the severe systemic inflammation and insulin resistance inherent to RA.

Serum vaspin is a valuable prognostic biomarker for patients with acute myocardial infarction (AMI). While high levels are found in active RA as the rs2236242 T allele is linked to higher vaspin levels and RA susceptibility [20], low circulating levels independently predict a significantly higher risk of heart failure and recurrent infarction, and adding vaspin to traditional cardiovascular risk models statistically improves their accuracy in forecasting MACE [17]. Another recent study has demonstrated that compared to the healthy baseline, patients presenting with AMI demonstrated a significant depletion of serum vaspin (*p* < 0.001) [32].

### 6.2. Nesfatin-1: The Hypothalamic-Articular Link

Originally identified as a hypothalamic neuropeptide regulating satiety, nesfatin-1 is also secreted by peripheral adipose tissue and joint tissues. Nesfatin-1 levels are significantly higher in RA serum and synovium, correlating positively with IL-1β, TNF-α, and rheumatoid factor (RF) [18]. It promotes the migration of monocytes via the CCL2 (MCP-1) pathway, driving synovitis [33].

Despite its inflammatory role in the joint, some data suggest a protective role in the vasculature, representing a cardiovascular paradox. Studies have found that higher nesfatin-1 levels in RA are associated with a reduced risk of atherosclerotic plaque and lower cIMT [29]. This tissue-specific dichotomy, destructive in the joint but protective in the vessel, highlights the complexity of adipokine signaling and suggests that nesfatin-1 may influence lipid metabolism or plaque stability via pathways distinct from systemic inflammation.

### 6.3. Progranulin (PGRN)

Progranulin acts as a direct antagonist to TNF receptors (TNFR), theoretically providing anti-inflammatory effects. Serum progranulin is significantly elevated in RA patients with high disease activity, often paralleling TNF-α levels. A serum cutoff of 47.7 ng/mL has been proposed to predict high disease activity with significant sensitivity [19].

Mechanistic studies indicate that progranulin protects against cardiorenal injury by negatively regulating Wnt/β-catenin signaling, a pathway implicated in endothelial dysfunction and heart failure [34]. However, its clinical utility as a standalone CV predictor is complicated by its strong correlation with the general inflammatory burden.

### 6.4. Apelin and the APJ System

Apelin is a potent positive inotrope and vasodilator that signals through the APJ G-protein coupled receptor. Unlike most inflammatory adipokines, apelin levels are consistently decreased in RA patients [20].

The apelin–APJ axis is crucial for maintaining endothelial integrity and myocardial contractility. The suppression of apelin in RA compromises endothelial repair mechanisms and promotes vasoconstriction and myocardial stiffness. Reduced apelin levels correlate with increased Matrix Metalloproteinase-9 (MMP-9) activity, which facilitates plaque instability and rupture [35].

Restoring apelin signaling is being investigated as a strategy to treat HFpEF, a common comorbidity in RA, potentially addressing both the vascular and myocardial components of RA heart disease [36].

## 7. Therapeutic Modulation of the Adipokine Profile

The introduction of targeted synthetic DMARDs (tsDMARDs), specifically Janus kinase (JAK) inhibitors, alongside biological DMARDs (bDMARDs), has profoundly altered the metabolic landscape of RA management. Understanding the differential effects of these drugs on adipokines is essential for interpreting cardiovascular risk in treated patients. Table 2 summarizes the potential effects of RA therapies on adipokine profiles and cardiovascular risk.

### 7.1. The JAK Inhibitor Era (Tofacitinib, Baricitinib, Upadacitinib, Filgotinib)

JAK inhibitors have been scrutinized for their cardiovascular safety following the ORAL Surveillance trial, which showed a higher risk of MACE and VTE with tofacitinib compared to TNF inhibitors in older, high-risk patients. A key metabolic feature of JAK inhibitor therapy is the “lipid paradox reversal,” a reversible increase in LDL and HDL cholesterol levels. Recent evidence suggests this lipid rise is accompanied by a stabilization of the adipokine profile.

In a one-year follow-up study, tofacitinib treatment significantly increased serum leptin and adipsin levels while decreasing chemerin and myeloperoxidase (MPO). Notably, adiponectin and resistin levels remained stable [38]. The increase in leptin alongside a decrease in inflammation (CRP) may indicate a “decoupling” of leptin from its downstream inflammatory effects due to the blockade of the JAK-STAT pathway.

Although JAK inhibitors can reduce inflammation and modify circulating lipid and adipokine concentrations, these biological changes cannot be assumed to translate into lower rates of clinical cardiovascular events. In ORAL Surveillance, a randomized safety study of patients with RA aged at least 50 years and with an additional cardiovascular risk factor, tofacitinib was associated with higher rates of major adverse cardiovascular events than TNF inhibitors [37]. Thus, the cardiovascular implications of JAK inhibition must be interpreted in the context of baseline cardiovascular and thromboembolic risk rather than inferred from lipid or adipokine changes alone.

The increase in circulating cholesterol after suppression of systemic inflammation may partly reflect reversal of the RA-associated lipid paradox; however, this metabolic explanation does not negate the observed cardiovascular and thrombotic safety signals with specific therapies. Treatment selection should include the patient’s previous cardiovascular disease or VTE, age, smoking status, cardiometabolic conditions, and other relevant clinical risk factors [37].

### 7.2. Biologics: TNF and IL-6 Inhibitors

TNF Inhibitors such as adalimumab and etanercept have the most robust data for adipokine modulation. Treatment leads to a significant increase in total and HMW adiponectin and a decrease in resistin and chemerin. This restoration of the adiponectin/leptin balance is strongly correlated with improved endothelial function and reduced arterial stiffness [29].

Moreover, by directly blocking the IL-6 receptor, tocilizumab profoundly impacts the hepatic production of CRP and alters adipocyte signaling. It increases adiponectin levels, though potentially less robustly than TNF inhibitors regarding the HMW isoform. However, its potent suppression of systemic inflammation makes it highly effective in reducing the “inflammatory” component of CV risk.

### 7.3. Glucocorticoids

While effective for joint symptoms, glucocorticoids worsen the metabolic profile by promoting insulin resistance, central obesity, and increasing leptin levels while suppressing osteocalcin and adiponectin. This adverse metabolic shift underscores the importance of minimizing steroid use to reduce long-term cardiovascular risk [29].

## 8. Integrated Risk Stratification: Adipokines vs. Traditional Scores

The evidence reviewed suggests that single biomarkers are unlikely to capture the complexity of the RA phenotype. Multimarker approaches integrating adipokines, cardiac biomarkers, and inflammatory measures require formal clinical validation.

### 8.1. Multi-Marker Panels

Proposed metabolic-inflammatory risk panels combine investigational adipokines with conventional cardiac and inflammatory measures. Elevated leptin, chemerin, and resistin have been associated with features of an atherogenic phenotype, whereas higher adiponectin together with NT-proBNP may accompany cardiac strain or a catabolic phenotype [11]. Lower omentin and apelin concentrations have been associated with endothelial dysfunction. However, available associations are heterogeneous, and no adipokine panel has been adequately externally validated, calibrated, or shown to improve clinical outcomes when used to guide care. Consequently, adipokine profiling should be considered a research approach rather than a component of routine cardiovascular risk assessment. Statins, antihypertensive agents, and antithrombotic treatment should not be initiated solely on the basis of an adipokine pattern [9].

### 8.2. Sex-Specific Differences in Risk

A 2026 comparative study highlighted profound sex differences in metabolic risk profiles among RA patients. Metabolic syndrome (MetS) is significantly more prevalent in female RA patients (56%) compared to males (36.7%) (*p* < 0.001) [39]. In women, MetS components, driven by visceral adiposity and adipokine dysregulation (high leptin, low omentin), disproportionately elevate CV risk, effectively nullifying the hormonal cardioprotection typically seen in pre-menopausal women. On the other hand, male RA patients exhibit higher baseline cardiovascular risk scores (SCORE-2) but are less likely to have the specific “adipo-metabolic” cluster seen in women. Hence, adipokine profiling may be particularly high-yield in female patients to detect “metabolically obese” individuals who have normal BMIs but high visceral adipose activity and hidden CV risk.

### 8.3. Correlations with Imaging Biomarkers

Adipokine levels correlate strongly with structural surrogates of cardiovascular disease, offering a “liquid biopsy” of the vessel wall. Carotid Intima-Media Thickness (cIMT) positively correlates with leptin, resistin, and chemerin; negatively with omentin [16]. Moreover, pulse wave velocity (PWV), a marker of arterial stiffness, positively associated with chemerin and resistin [40]. Furthermore, coronary artery calcium (CAC) is associated with low omentin and altered visfatin/nesfatin-1 ratios [28].

## 9. Discussion: Systemic RA, Cardiometabolic Risk, and Clinical Implications

### 9.1. Inflammatory Burden and Extra-Articular Disease

The evidence reviewed in this article supports an integrated interpretation of cardiovascular risk in RA. Persistent inflammation can contribute to endothelial activation, metabolic dysregulation, and myocardial and vascular injury alongside conventional risk factors. Extra-articular manifestations reinforce the concept of RA as a systemic disease rather than a disorder confined to synovial tissue [3]. Disease activity, disease duration, and cumulative inflammatory exposure may affect both circulating adipokines and clinical cardiovascular outcomes, creating potential confounding and limiting causal interpretation of observational biomarker associations. Consequently, adipokines should be considered candidate indicators of an inflammatory–metabolic state rather than proven independent drivers or ready-to-use clinical tests.

### 9.2. Venous Thromboembolism and Treatment-Related Risk

In addition to myocardial infarction, stroke, and heart failure, VTE warrants explicit consideration in RA. The real-world cohort and accompanying literature appraisal by Conforti et al. support the established clinical association between RA and venous thromboembolic events [7]. By contrast, evidence that particular adipokines independently predict VTE after comprehensive clinical adjustment is limited, and mechanistic associations should not be described as validated predictive tests. Selected JAK inhibitor safety findings also require attention to baseline thromboembolic risk [37]. Atherosclerotic risk scores, including SCORE-based tools, do not estimate deep-vein thrombosis or pulmonary embolism risk. Clinical evaluation of prior VTE, immobility, recent surgery, obesity, active disease, and relevant medication exposure should therefore be individualized. Neither RA diagnosis nor an elevated adipokine concentration alone justifies routine anticoagulant prophylaxis.

### 9.3. Cardiometabolic Multimorbidity, Covariates, and Evidence Limitations

The coexistence of hypertension, type 2 diabetes, and dyslipidemia may identify a clinically more complex RA phenotype than consideration of each risk factor in isolation. In the 757-patient cross-sectional study by Ruscitti et al., 13.5% had cardiometabolic multimorbidity [8]. Relative to participants without multimorbidity, extra-articular manifestations were more frequent (17.7% vs. 8.2%), clinical remission was less frequent (8.5% vs. 15.7%), and a history of bDMARD failure was more frequent (78.4% vs. 40.4%). These findings strengthen the connection between inflammatory–metabolic pathways and disease burden, particularly in older patients or those with prolonged treatment exposure. Nevertheless, because the study was observational and cross-sectional, causal direction and treatment effects cannot be inferred; obesity, smoking, age, disease duration, and glucocorticoid exposure remain important potential confounders.

This article is a narrative literature review and does not include an original patient cohort, newly collected clinical variables, or a pooled patient-level statistical analysis. Accordingly, prior cardiovascular disease or VTE, hypertension, diabetes, dyslipidemia, obesity, and smoking were not recorded by the authors as baseline variables of a newly recruited study population. The primary studies cited in this review differed in the availability, definition, reporting, and statistical adjustment of these covariates; the present review did not conduct a uniform study-by-study extraction or quantitative confounder-adjusted synthesis. As a result, apparent independent prognostic effects of adipokines may be influenced by residual confounding, reverse causation, treatment exposure, and differences in outcome ascertainment. This methodological limitation should be considered when interpreting associations with clinical events or imaging surrogates.

### 9.4. Implications for Cardiovascular Screening and Routine Practice

In routine practice, established cardiovascular prevention and control of RA disease activity remain the priorities. The 2015/2016 EULAR recommendations support regular cardiovascular risk evaluation, at least once every five years and following important antirheumatic treatment changes, alongside management of conventional risk factors [9]. When appropriate for the selected general-population algorithm, the EULAR 1.5 multiplication factor may be considered according to those recommendations [9]. History of cardiovascular disease and VTE, hypertension, diabetes, dyslipidemia, body mass index, smoking, and medication exposure should inform individualized assessment. Arterial and venous risks require different clinical approaches. Although carotid imaging and adipokine profiling are research interests, biomarker measurement cannot presently replace established cardiovascular risk scores, guideline-based preventive therapy, or treatment-specific safety evaluation.

Prospective studies are needed to establish whether adipokines add reproducible prognostic value beyond age, sex, smoking, blood pressure, diabetes, lipid measurements, obesity, established cardiovascular disease, disease activity, and antirheumatic treatment exposure. Such studies should distinguish incident arterial events, heart failure, and VTE, test external validity, and assess whether biomarker-guided management improves patient outcomes rather than only surrogate measures.

## 10. Conclusion and Future Directions

The assessment of cardiovascular risk in rheumatoid arthritis has evolved from a simple monitoring of lipids and blood pressure to a sophisticated evaluation of the immuno-metabolic landscape. Adipokines are the functional units of this landscape, translating systemic inflammation into vascular pathology.

Overall, available evidence supports the biological relevance of adipokines to the inflammatory and cardiometabolic milieu of RA, but does not establish them as routine prognostic tests or proven therapeutic targets. Adiponectin has context-dependent associations, including with heart failure and catabolic states; leptin, resistin, visfatin, and chemerin may reflect or participate in vascular inflammatory processes, while omentin and apelin have been explored as potential protective mediators. Associations with clinical outcomes, carotid intima-media thickness, and vascular stiffness remain vulnerable to confounding and require independent prospective validation. In particular, the elevated VTE burden in RA is clinically relevant, but the incremental predictive value of individual adipokines for VTE has not been established.

Although antirheumatic therapies can modify inflammatory and metabolic biomarkers, these changes must not be equated with proven cardiovascular protection; available MACE and VTE safety findings should inform treatment selection [37]. The coexistence of cardiometabolic conditions may identify patients with more complex RA manifestations and treatment histories [8]. Until prospective studies demonstrate reliable incremental prediction and clinical utility, adipokine profiling should remain investigational. Systematic cardiovascular prevention, appropriate consideration of VTE risk, and optimal control of RA inflammation are the principal clinical priorities [9].

## Figures and Tables

**Table 1:** Summary of Adipokine Roles in RA Cardiovascular Risk.

Adipokine	Role in RA CVD	Key Mechanism	Predictive Value
Adiponectin	Paradoxical Risk Factor	Marker of cachexia/HF; induces synovial VEGF	High levels predict mortality & HF hospitalization [11]
Leptin	Pro-Atherogenic	Oxidative stress; platelet aggregation; PAI-1 induction	Predicts CVD mortality & VTE risk [11]
Resistin	Synergistic Inflammatory Risk	TLR4/CAP1 binding (endothelial activation); Myocardial fibrosis (inhibits AMPK)	Predicts incident Heart Failure (HR 1.44); Strong CIMT correlation (highest in RA + MetS); [12] Did not predict TBR change in TARGET trial [13]
Visfatin (NAMPT)	Plaque Destabilizer	MMP induction (plaque rupture); NAD+ salvage; Angiogenesis	High levels linked to unstable plaque/MACE; Low serum levels paradoxically linked to joint erosions (sequestration) [14]
Chemerin	Endothelial Activator	Recruitment of DC/Macrophages via ChemR23	Predicts stroke, plaque instability, & cIMT [15]
Omentin	Lost Protector	AMPK activation; eNOS phosphorylation	Inverse correlation with calcification & arterial stiffness [16]
Vaspin	Compensatory Marker	Insulin sensitization; anti-apoptotic	Significant prognostic marker of MACE in AMI patients [17]
Nesfatin-1	Controversial	Synovial inflammation vs. vascular protection	High synovial levels; variable CV data [18]
Progranulin	Inflammatory Marker	TNFR antagonism; Wnt signaling regulation	Predicts high disease activity [19]
Apelin	Vascular Guardian	Inotropy; vasodilation; endothelial repair	Depletion predicts HFpEF & endothelial dysfunction [20]

**Table 2:** Impact of RA Therapies on Adipokine Profiles and CV Risk.

Therapy Class	Adiponectin	Leptin	Resistin	Visfatin	Chemerin	Omentin	Net CV Mechanism
TNF Inhibitors	Increase (Restores HMW)	Decrease/ Neutral	Decrease	Decrease	Decrease	Increase	Improved endothelial function; plaque stabilization [1]
IL-6 Inhibitors	Increase	Decrease	Decrease	Decrease	Decrease	Neutral	Reduction in inflammatory atherogenesis; lipid elevation [25]
JAK Inhibitors	Neutral/Increase	Increase (Decoupled)	Stable	Variable	Decrease	Neutral	Biomarker/lipid changes do not establish cardiovascular safety; assess MACE/VTE risk [37]
Glucocorticoids	Decrease	Increase	Increase	Variable	Increase	Decrease	Worsens metabolic syndrome; promotes visceral adiposity [6]
Methotrexate	Neutral	Neutral	Neutral	Neutral	Neutral	Neutral	General anti-inflammatory benefit; accumulation of adenosine [1]

## Data Availability

No new data were created or analyzed in this study. Data sharing is not applicable to this article.

## Abbreviations

The following abbreviations are used in this manuscript:

AMI	Acute Myocardial Infarction
AMPK	AMP-activated Protein Kinase
CAP1	Adenylyl Cyclase-Associated Protein 1
CIMT	Carotid Intima-Media Thickness
CVD	Cardiovascular Disease
DC	Dendritic Cells
eNOS	Endothelial Nitric Oxide Synthase
HF	Heart Failure
HFpEF	Heart Failure with Preserved Ejection Fraction
MACE	Major Adverse Cardiovascular Events
MetS	Metabolic Syndrome
MMP	Matrix Metalloproteinase
PAI-1	Plasminogen Activator Inhibitor-1
TBR	Target-to-Background Ratio
TLR4	Toll-like Receptor 4
TNFR	Tumor Necrosis Factor Receptor
VEGF	Vascular Endothelial Growth Factor
VTE	Venous Thromboembolism
TNF	Tumor Necrosis Factor
IL-6	Interleukin-6
JAK	Janus Kinase
CV	Cardiovascular
HMW	High Molecular Weight
